# *Citrus medica* “Otroj”: Attenuates Oxidative Stress and Cardiac Dysrhythmia in Isoproterenol-Induced Cardiomyopathy in Rats

**DOI:** 10.3390/nu5114269

**Published:** 2013-10-28

**Authors:** Mohammed A. Al-Yahya, Ramzi A. Mothana, Mansour S. Al-Said, Kamal Elddin El-Tahir, Mohammed Al-Sohaibani, Syed Rafatullah

**Affiliations:** 1 Department of Pharmacognosy, College of Pharmacy, King Saud University, P.O. Box 2457, Riyadh 11451, Saudi Arabia; E-Mails: alyahya@ksu.edu.sa (M.A.A.); msalsaid@ksu.edu.sa (M.S.A.); 2 Medicinal, Aromatic and Poisonous Plants Research Center (MAPPRC), College of Pharmacy, King Saud University, P.O. Box 2457, Riyadh 11451, Saudi Arabia; E-Mail: srafat@ksu.edu.sa; 3 Department of Pharmacology, College of Pharmacy, King Saud University, P.O. Box 2457, Riyadh 11451, Saudi Arabia; E-Mail: keltahir5@gmail.com; 4 Department of Pathology, King Khalid University Hospital, King Saud University, P.O. Box 2925, Riyadh 11461, Saudi Arabia; E-Mail: sohibani@ksu.edu.sa

**Keywords:** cardioprotective, *Citrus medica*, antioxidant, oxidative stress, lipid peroxidation, cardiac dysrhythmia

## Abstract

*Citrus medica* L. commonly known as Otroj, is an important medicinal plant reputed for its nutritious and therapeutic uses. The present work was undertaken to investigate the protective effect of the ethanolic extract of otroj (EEOT) against isoproterenol (ISO)-induced cardiotoxicity in rats. In addition, the antioxidant activity and the phenolic and flavonoidal contents were determined. Rats were administered EETO (250 and 500 mg/kg) or vehicle orally for 15 days along with ISO (85 mg/kg, s.c.) on the 14th and 15th day. ISO induced cardiac dysfunction, increased lipid peroxidation and alteration of myocyte-injury specific marker enzymes. ISO also showed an increase in levels of plasma cholesterol, triglycerides (TG), LDL-C, and VLDL-C. Moreover, the histological investigations showed myocardial necrosis and inflammation. EETO treatment brought the above parameters towards normal level. Moreover, *in vitro* DPPH radical scavenging and β-carotene-linoleic acid tests of the EEOT exhibited a notable antioxidant activity in both assays used. In addition, histopathological examination reconfirmed the protective effects of EEOT. Thus, the present study reveals that *C. medica* alleviates myocardial damage in ISO-induced cardiac injury and demonstrates cardioprotective potential which could be attributed to its potent antioxidant and free radical scavenging activity.

## 1. Introduction

According to an earlier estimation of the World Health Organization (WHO), about 17 million people die every year due to cardiovascular disease (CVD) [1]. Cardiovascular diseases are directly or indirectly responsible for oxidative damage, which causes an insufficient blood supply to the myocardium, leads to myocardial infarction (MI) or heart attack [2]. Despite improvement in clinical care and better awareness, MI still remains the leading cause of mortality in the world [3].

Isoproterenol (ISO), a synthetic nonselective β-adrenoceptor agonist is well accepted rat model to induce myocardial infarction in order to evaluate several cardiac dysfunctions in laboratory animals. The pathophysiological and morphological changes of ISO-induced myocardial changes are similar to those observed in human with MI [4].

*Citrus medica* L. “Otroj” (Brain citron), is a member of Rutaceae family. *Citrus* fruits in general have long been known to contain many important nutritious components such as phenolics, flavanones, ascorbic acid (vitamin C), and pectin, which are recognized as potent antioxidants [5]. Previously and reported that several species of *Citrus* peels have exerted antioxidant effects, has been reported to possess stress-induced antipeptic ulcer potential [6]. Earlier, *Citrus medica* peel extract has shown potent anti-inflammatory and pain reducing activity in rats [5]. In Arab traditions, Unani and Ayurvedic medicine, otroj peel and whole fruit are used as carminative, stomachic, cardiotonic, refrigerant, and as an appetizer. Moreover, it has been described that otroj is beneficial in the induration of the spleen tumors [7]. However, there is no scientific report available in the literature about the *Citrus medica* whole fruit (which is consumed in traditional medicine) on its usefulness in heart ailments. Therefore, in the present investigation, an attempt has been made to explore the possible *in vivo* cardioprotective and *in vitro* antioxidant potential of ethanol extract of the whole fruit of *Citrus medica* in rats against isoproterenol-induced cardiac disturbances.

## 2. Experimental Section

### 2.1. Animals

Male Wistar albino rats, 12 weeks of age, weighing 150–170 g, obtained from the Experimental Animal Care Center, College of Pharmacy, King Saud University, Riyadh, Saudi Arabia were used in the experiments. The animals were kept at a constant temperature (22 ± 2 °C), humidity (55%) and light-dark conditions (12/12 h light/dark ratio). The animals were provided with Purina chow diet and drinking water *ad libitum.* The conduct of experiments and the procedure of sacrifice (using ether) were approved by the Ethics Committee of the Experimental Animal Care Center, College of Pharmacy, King Saud University, Riyadh, Saudi Arabia.

### 2.2. Plant Material and Preparation of Extract

The fruits of *Citrus medica* “otroj” were purchased from the local vegetable market in Riyadh and its identity was confirmed by Dr. M. Yusuf, Taxonomist of the Department of Pharmacognosy, College of Pharmacy, King Saud University, where a voucher specimen (No. 7262) of the sample has been kept in the Herbarium. The otroj fruits were cut into small pieces and dried in the drying room. The dried whole fruit (500 g) was coarsely powdered and macerated in 3 L of 96% ethanol for 72 h using the percolation method. The solvent was then removed at 40 °C under reduced pressure in a rotavapor. The ethanolic extract of otroj (EEOT) was then suspended in distilled water just before its administration to the animals.

### 2.3. Acute Toxicity Test

The acute toxicity of the ethanol extract of the whole fruit of *Citrus medica* was evaluated in rats. Six female rats (weight: 180–200 g) received a single high dose of EEOT (2 g/kg orally) by gavage. The animals were observed for toxic symptoms continuously for the first 4 h after dosing. Finally, the number of survivors was noted after 24 h and these animals were then maintained for a further 13 days with daily observation [8].

### 2.4. Induction of Myocardial Disturbance

Experimental myocardial disturbance was induced by injecting isoproterenol hydrochloride (ISO) (dissolved in physiological solution) subcutaneously to rats at a dose of 85 mg/kg daily for two consecutive days [9]. To test the effect of *Citrus medica* extract on isoproterenol-induced cardiac disturbances, the animals were divided into four groups. Group 1 was the control administered saline only and group two was administered isoproterenol. Groups three and four were treated with *Citrus medica* alcoholic extract in doses of 250 and 500 mg/kg, respectively, for 15 days. Isoproterenol was administered (s.c.) on days 14 and 15. The animals were then anaesthetized with urethane (1.25 g/kg i.p) after the second dose of isoproterenol on day 15 and prepared for measurement of the ECG (Progetti ECG machine, Trofarello, Italy) from lead II as described before [10].

### 2.5. Preparation of Cardiac Tissue Homogenate

At the end of the ECG recordings, blood was removed from the orbital sinuses to perform the biochemical test and then the animals were operated surgically to remove the hearts, which were washed with ice cold saline and homogenized in 0.15 M KCl solution in water. Heart tissues were immediately removed and washed with ice cold saline and homogenized in the appropriate buffer in a tissue homogenizer.

### 2.6. Estimation of Marker Enzymes

Levels of plasma alanine aminotransferase (ALT), aspartate aminotransferase (AST) [11], lactate dehydrogenase (LDH) [12], and creatine kinase (CK) [13] were then estimated using Reflotron^®^ Plus Analyzer and Roche Diagnostic Kits (Roche Diagnostics GmbH, Mannheim, Germany).

### 2.7. Estimation of Lipid Profile

The total cholesterol (TC) [14], triglycerides (TG) [15], and high-density lipoproteins (HDL-C) [16] were estimated in plasma using the Refloton instrument of the specific kits (Roche Diagnostics GmbH, Mannheim, Germany).

### 2.8. Lipid Peroxidation (LPO) Determination

The method reported by Utley *et al.* [17] was followed. The heart tissue was homogenized in 0.15 M KCl (at 4 °C, Potter-Elvehjem type C homogenizer) to give a 10% w/v homogenate. Aliquots of homogenate (1 mL) were incubated at 37 °C for 3 h in a metabolic shaker. Following this, 1 mL of 10% aqueous trichloroacetic acid (TCA) was added and mixed. The mixture was then centrifuged at 800 g for 10 min. Following this, supernatant (1 mL) was mixed with 1 mL of 0.67% thiobarbituric acid and placed in a boiling water bath for 10 min. The mixture was cooled and diluted with 1 mL distilled water. The absorbance of the solution was then read using spectrophotometer (UVmini-1240, Shimadzu Italia, Milano, Italy) at 532 nm. The content of malondialdehyde (MDA) (nmol/g wet tissue) was then calculated, by reference to a standard curve of MDA solution.

### 2.9. Estimation of Non-Protein Sulfhydryl Groups (NP-SH)

Cardiac NP-SH was measured according to the method of Sedlak and Lindsay [18]. The heart was homogenized in ice-cold 0.02 M ethylene diamine tetraacetic acid (EDTA). Aliquots of 5 mL of the homogenates were mixed in 15 mL test tubes with 4 mL of distilled water and 1 mL of 50% TCA. The tubes were shaken intermittently for 10 min and centrifuged at 3000 rpm. Two milliliters of supernatant were mixed with 4 mL Tris buffer (0.4 mol/L, pH 8.9) and 0.1 mL of 5,5ʹ-dithio-*bis*(2-nitrobenzoic acid) (DTNB) and the sample was shaken. The absorbance was read within 5 min of addition of DTNB at 412 nm against a reagent blank.

### 2.10. Determination of Total Protein (TP)

The TP was estimated by the kit method, supplied by Crescent Diagnostics, Jeddah, Saudi Arabia. The absorbance of this complex at 546 nm is proportional to the protein concentration. The serum total protein was calculated using the equation:

Serum total protein = Abs_sample_/Abs_standard_ × concentration of standard (1)

### 2.11. Histopathological Studies

The heart tissues were fixed in 10% buffered formalin and processed using a VIP tissue processor. The processed tissues were then embedded in paraffin blocks and sections of about 5 μm thickness were cut by employing an American optical rotary microtome. These sections were stained with hematoxylin and eosin using routine procedures [19]. The slides were examined for pathomorphological changes.

### 2.12. Studies of the *in Vitro* Antioxidant Activity

#### 2.12.1. Scavenging Activity of DPPH Radical

The radical scavenging ability of the EEOT against DPPH was evaluated as previously described [20]. In the presence of an antioxidant which can donate an electron to DPPH, the purple color, typical for free DPPH radical decays, and the change in absorbency at λ = 517 nm was measured. The test provides information on the ability of a compound to donate a hydrogen atom, on the number of electrons a given molecule can donate, and on the mechanism of antioxidant action. The extract was redissolved in methanol and various concentrations (10, 50, 100, 500, and 1000 μg/mL) of the extract, 125 μL prepared DPPH (1 mM in methanol) and 375 μL solvent (methanol) were added. After 30 min incubation at 25 °C, the decrease in absorbance was measured at λ = 517 nm. The radical scavenging activity was calculated from the equation:

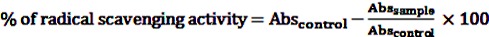
(2)

#### 2.12.2. β-Carotene-Linoleic Acid Assay

The antioxidant activity of the crude extract was evaluated using the β-carotene bleaching method described and modified by Mothana [21]. One milliliter of a 0.2 mg/mL β-carotene solution in chloroform was added to flasks containing 0.02 mL of linoleic acid and 0.2 mL of Tween-20. The chloroform was removed at 40 °C using a rotary evaporator. The resultant mixture was immediately diluted with 100 mL of distilled water and mixed for 1–2 min to form an emulsion. A mixture prepared similarly but without β-carotene, was used as a blank. A control containing 0.2 mL of 80% (v/v) methanol instead of extract was also prepared. A 5 mL aliquot of the emulsion was added to a tube containing 0.2 mL of the sample extract at 1 mg/mL. Rutin (1 mg/mL) was used as a standard. The tubes were placed in a water bath at 40 °C for 2 h. Absorbance was read at 470 nm at 15 min intervals. The antioxidant activity was calculated using the equation:

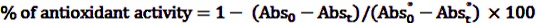
(3)
where, 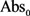 and 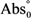 are the absorbance values measured at zero time of incubation for sample extract and control, respectively.


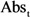
 and 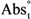 are the absorbance values for sample extract and control, respectively, at *t* = 120 min.

### 2.13. Total Phenolic Content

The Folin-Ciocalteu method was used to determine the total phenolic content (TPC) of the extract according to [22]. Values of TPC were estimated by comparing the absorbance of each sample with a standard response curve generated using gallic acid (0, 12.5, 25, 50, 100, and 200 µg/mL). The results were expressed as mg gallic acid equivalents (GAE)/100 g of the extract. All the measurements were taken in triplicate and the mean values were calculated.

### 2.14. Total Flavonoid Content

The total flavonoid content was determined by using a colorimetric assay according to Djeridane * et al.* [23]. Briefly, an aliquot of 1 mL of EETO solution or standard solution was mixed individually with the same volumes of solution of 2% aluminum chloride (AlCl_3_) allowed standing at room temperature for 10 min. The absorbance was then read at 415 nm. A calibration curve was prepared with quercetin and the results were expressed as mg quercetin equivalents (CE)/100 g of the extract.

### 2.15. Statistical Analysis

Values are given as arithmetic means ± standard error of the mean (S.E.M). The data were statistically analyzed by using a one-way analysis of variance (ANOVA), followed by Dunnett’s *t*-test.

## 3. Results

### 3.1. Acute Toxicity Test

No toxicity symptoms (e.g., convulsions, myosis, mydriasis, diarrhea, increasing respiration, urination, and muscle relaxation) were recorded. The LD_50_ value by oral route could not be determined as no lethality was observed up to 2.0 g/kg of the EEOT in the animals.

### 3.2. Effect of Ethanolic Extract of Otroj (EEOT) on Marker Enzymes

Table 1 represents the effect of the ethanolic extract of *Citrus medica* on cardiac marker enzymes (ALT, AST, LDH, and CK) of control and test rats. The subcutaneous administration of ISO developed a marked myocardiopathy, as evident from significant increase in plasma ALT, AST, LDH, and CK, as compared to normal (control) group. The elevated levels of these plasma markers were reduced in the animal groups treated with the EEOT in both doses used (250 and 500 mg/kg) (Table 1). Although, AST level was declined in the group treated with EEOT at 250 mg/kg dose, it was not found to be statistically significant.

### 3.3. Effect of EEOT on Lipid Profile

The levels TC, TG, HDL, LDL, and VLDL levels in serum of control and experimental groups of rats are shown in Table 2. Rats treated with ISO showed a significant increase in these levels, except that the HDL and VLDL levels were significantly decreased. Treatment of rats with EEOT caused profound decreases in the serum levels of TC, TG, LDL, and VLDL (Table 2). However, a significant elevation of HDL level was observed in the extract treated animals.

**Table 1:** Effect of *Citrus medica* (EEOT) on serum marker enzymes of control and experimental rats.

Treatment Group (*n* = 6)	AST (U/L)	ALT (U/L)	LDH (U/L)	CK (U/L)
Normal control	72.15 ± 2.34	29.28 ± 2.17	84.11 ± 2.62	139.50 ± 4.26
ISO (85 mg/kg)	166.66 ± 10.61 ***^, a^	92.13 ± 4.88 ***^, a^	131.77 ± 4.01 ***^, a^	198.50 ± 6.14 ***^, a^
EEOT (250 mg/kg) + ISO	161.16 ± 5.85 ^b^	84.25 ± 5.39 ^b^	118.45 ± 2.86 *^, b^	171.33 ± 3.58 **^, b^
EEOT (500 mg/kg) + ISO	137.00 ± 3.44 *	69.11 ± 3.08 **^, b^	107.22 ± 3.38 ***^, b^	151.50 ± 3.38 ***^, b^

The results are expressed as mean ± SD of six rats * *p* < 0.05; ** *p* < 0.01; *** *p* < 0.001; ANOVA, followed by Dunnett’s multiple comparison test. ^a^ As compared with normal group; ^b^ As compared with only ISO only group.

**Table 2:** Effect of EEOT on serum lipid metabolism and serum lipoproteins of control and experimental rats.

Treatment Group (*n* = 6)	Cholesterol (mg/dL)	Triglycerides (mg/dL)	HDL-C (mg/dL)	LDL-C (mg/dL)	VLDL-C (mg/dL)
Normal control	108.84 ± 5.83	58.66 ± 4.66	51.14 ± 2.88	45.96 ± 5.08	11.73 ± 0.93
ISO (85 mg/kg)	225.17 ± 9.74 ***	121.51 ± 5.46 ***^,a^	29.68 ± 2.17 ***^,a^	171.18 ± 9.14 ***^,a^	24.30 ± 1.09 ***^,a^
EEOT (250 mg/kg) + ISO	189.11 ± 6.72 *	110.54 ± 6.15 ^b^	42.01 ± 3.65 *^,b^	124.99 ± 9.65 **^,b^	22.10 ± 1.23 ^b^
EEOT (500 mg/kg) + ISO	157.82 ± 6.38 ***	69.19 ± 3.61 ***^,b^	40.18 ± 2.41 **^,b^	103.79 ± 6.21 ***^,b^	13.83 ± 0.72 ***^,b^

The results are expressed as mean ± SD of six rats, * *p* < 0.05; ** *p* < 0.01; *** *p* < 0.001; ANOVA, followed by Dunnett’s multiple comparison test. ^a^ As compared with normal group; ^b^ As compared with only ISO only group.

### 3.4. Effect of EEOT on MDA, NP-SH, and TP

**Figure 1:**
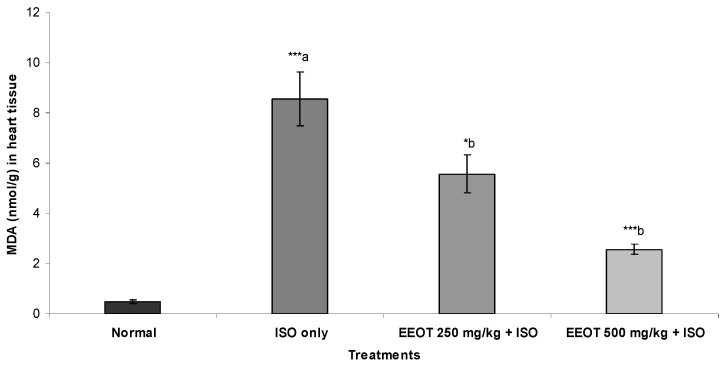
Effect of EEOT on the concentration of malondialdehyde (MDA) in the heart tissue of the rats treated with isoproterenol.

**Figure 2:**
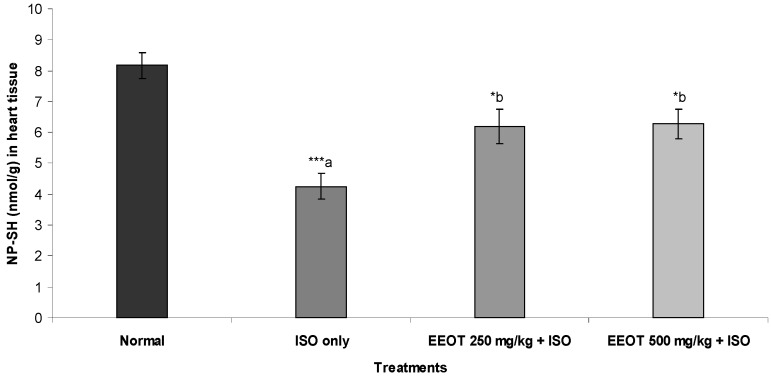
Effect of EEOT on the level of nonprotein sulfhydryl (NP-SH) in the heart tissue of the rats treated with isoproterenol.

**Figure 3:**
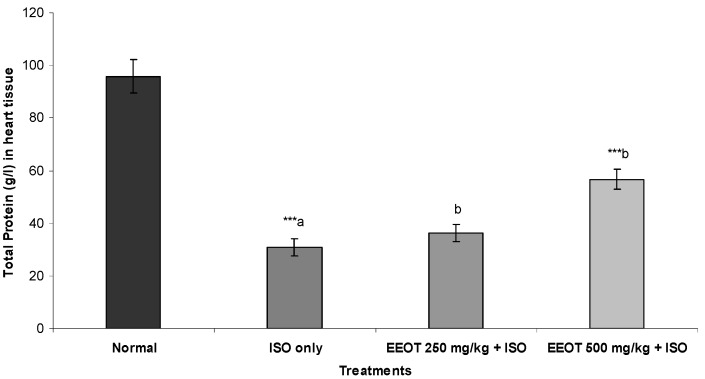
Effect of EEOT on the level of total protein (T.P.) in the heart tissue of the rats treated with isoproterenol.

The results also indicated that injection of ISO resulted in a significant elevation of MDA and a significant reduction in NP-SH and TP concentration in heart tissue (Figure 1, Figure 2 and Figure 3). Treatment of rats with EEOT caused a significant attenuation in MDA contents and elevated the NP-SH and TP levels in heart muscle (Figure 1, Figure 2 and Figure 3).

All values represent mean ± SEM. * *p* < 0.05; *** *p* < 0.001; ANOVA, followed by Dunnett’s multiple comparison test; ^a^ As compared with normal group; ^b^ As compared with only ISO group.

All values represent mean ± SEM. * *p* < 0.05; *** *p* < 0.001; ANOVA, followed by Dunnett’s multiple comparison test; ^a^ As compared with normal group; ^b^ As compared with only ISO only group.

All values represent mean ± SEM. *** *p* < 0.001; ANOVA, followed by Dunnett’s multiple comparison test; ^a^ As compared with normal group; ^b^ As compared with only ISO group.

### 3.5. Effect of EEOT on Histopathological Evaluation

**Figure 4:**
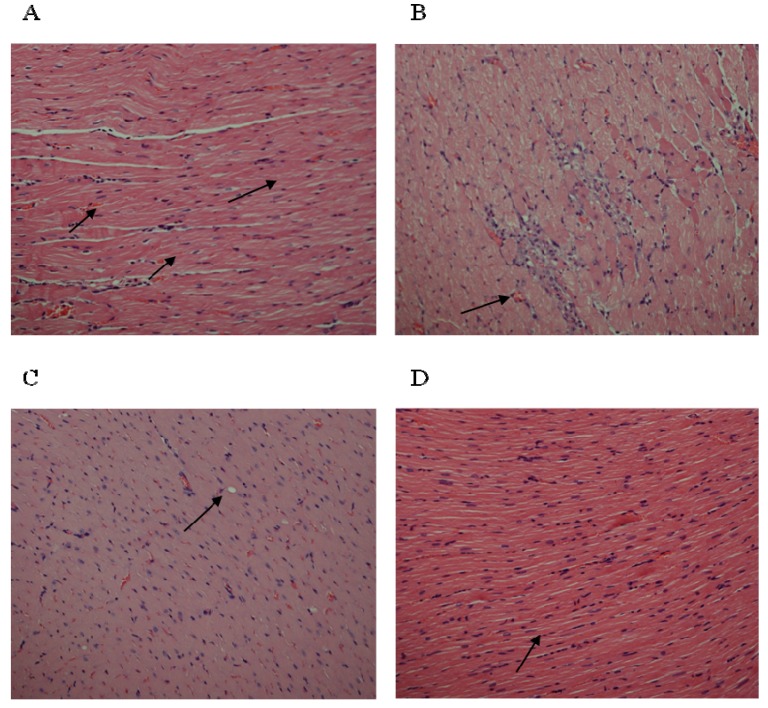
Light micrographs showing the effect of *Citrus medica* extract on myocardial cells. (**A**) normal rats (control group) showing normal myocardial cells, H & E. 200×; (**B**) histological changes after ISO only treated rats (group II) showing inflammation including some macrophage with mitotic figures in myocardial cells indicating myocardial injury and ischemia, H & E. 200×; (**C**) (250 mg/kg) + ISO group showing mild enlargement with some damage to myofiber at lower dose of EEOT, H & E. 200×; (**D**) (500 mg/kg) + ISO group showing no inflammation, mild residual cardiac muscle fiber injury, H & E. 200×.

Histopathological assessment on the other hand, revealed that in the control group, the myocardium of rats had normal cardiac tissue fibers (Figure 4A). The histomorphology of the heart muscle in the ISO only treated group were characterized by chronic inflammation, indicating some macrophage with mitotic figures in some myocardial cells, indicating myocardial and ischemia (Figure 4B). Effect of treatment of rats with EEOT at (250 mg/kg/day) dose plus ISO group was mild nuclear enlargement with some damage to myofiber (Figure 4C). Rats pretreated with EEOT + ISO (500 mg/kg/day) showed no inflammation, mild residual muscle fiber injury (Figure 4D).

### 3.6. Effect of EEOT on Cardiac Rhythm

Treatment of normal rats with isoproterenol at doses of 85 mg/kg s.c., for two days, significantly increased the heart rate from a basal level of 280 ± 11.4 beats/min to 430 ± 13.9 beats/min (*p* < 0.001, *n* = 6) with an increase of 53.5% ± 3.1%. Treatments of the animals with *Citrus medica* alcoholic extract at doses of 250 and 500 mg/kg, for 15 days, significantly suppressed isoproterenol induced cardiac disturbances and tachycardia. The mean heartbeats were 340 ± 17.3 and 330 ± 18.7, respectively (Table 3). The percentage effectiveness of the treatments in suppressing isoproterenol-induced tachycardia is shown in Table 3 (60% and 66%, respectively).

**Table 3:** Effect of EEOT on isoproterenol-induced cardiac tachycardia.

Treatment Group (*n* = 6)	% Increase in Heart Rate	% Effectiveness in Suppressing Isoproterenol-Induced Tachycardia
Isoproterenol (85 mg/kg) s.c.	53.35 ± 3.1	-
EEOT (250 mg/kg) + ISO	21.4 ± 2.1	60 ± 5.9 *
EEOT (500 mg/kg) + ISO	17.8 ± 2.5	66.7 ± 6.1 *

The results are expressed as mean ± SD of six rats, * *p* < 0.01; ANOVA, followed by Dunnett’s multiple comparison test.

### 3.7. Antioxidant Activity and Phenolic and Flavonoidal Contents

The results of the antioxidant activity are presented in Table 4. EEOT was able to reduce the stable free radical DPPH to the yellow-colored DPPH at low concentrations (100 and 500 μg/mL), almost near to ascorbic acid. Moreover, in the β-carotene/linoleic acid model system, the EEOT was also able to inhibit the discoloration of β-carotene at a concentration of 1 mg/mL. The total antioxidant value was 92% (Table 4). The observed antioxidant activities were comparable to that of the positive control, rutin (Table 4). Moreover, the EEOT showed high total phenol and flavonoidal values (192.4 ± 2.52 mg gallic acid equivalents/100  g and 74.1 ± 3.12 mg quercetin equivalents/100 g).

**Table 4:** Free radical scavenging activity, antioxidant activity and total phenolic and total flavonoidal contents of the EEOT.

Plant Species	Radical Scavenging Activity in (%)					Total Antioxidant Activity in (%)	TPC (mg GAE/100 g)	TFC (mg QE/100 g)
	10	50	100	500	1000	1000 (μg/mL)		
EEOT	13.1	39.0	72.9	87.0	93.5	92.8 ± 6.91	192.4 ± 2.52	74.1 ± 3.12
Ascorbic acid	19.5	71.2	85.5	92.7	94.1	-		
Rutin						93.1 ± 7.22		

TPC: Total phenolic content; TFC: Total flavonoidal content.

## 4. Discussion

The present investigation demonstrates the cardioprotective potential of the ethanolic extract of *Citrus medica* “otroj” (EEOT) in ISO-induced model of myocardiopathy in Wistar albino rats. Isoproterenol is known to produce cardiotoxic effects on the myocardium. The ISO-induced cardiac damage can be explained as generation of highly cytotoxic free radicals through autooxidation of catecholamine [24]. ISO-induced myocardial necrosis is well-accepted model of myocardial infarction in rats and the lesions produced by ISO, resemble to myocardial infarction in human subjects [25], and is a reliable rat model to study myocardial injury [26]. ISO caused an imbalance between oxygen supply and myocardial hyperactivity due to tachycardia [27]; increased cAMP and increased Ca^2+^ overload [28]. Another possible mechanism involved in myocardial pathogenesis is an increased oxidative stress and generation of free radicals due to metabolic products of ISO [29].

In our experiments, the ISO treatment caused a high level of diagnostic marker enzymes AST, ALT, LDH, and CK due to leakage takes place from tissue to blood serum due to damages or destroyed myocardial cells, because of insufficient supply of oxygen, the cell membrane become fragile or may rupture. The cellular enzymes in serum reflect the alteration in plasma membrane permeability [30]. The increased levels of these enzymes are indicative to severity of cell necrosis and ISO mediated peroxidative myocyte injury [31]. Pretreatment with EEOT significantly reduced the plasma enzyme levels, thereby restricting the leakage of these enzymes from myocardium. The restricted enzymes permeability might be due to the preservation of myofibrils and mitochondrial morphology, indicating cardioprotective activity of EEOT.

Myocardial infarction (MI) is also associated with altered lipid metabolism. Lipids play an important role in cardiovascular disease, by way of hyperlipidemia and thereby modifying the cellular membranes’ structure, composition and stability [32]. The increased concentration of cholesterol and building up in cardiac tissue has a definite affinity with cardiovascular injury [33]. It was observed in this study that the ISO administration caused a significant increase in the level of cholesterol and triglycerides in serum. When the rats were cotreated with EEOT, a decrease in both levels of cholesterol and TG was noted. There is mounting evidence that oxidative stress is involved in the pathogenesis of various disease conditions and generates reactive oxygen species (ROS). In the present study, the improvement in hyperlipidemia by EEOT may be due to significant reduction in lipolysis, and could possibly be due to its notable antioxidant and antilipid peroxidative property by protecting cardiac tissue membrane lipids from ISO-mediated lipid peroxidation [34]. However, the increased cholesterol level might be a result of diminished HDL-C, as HDL-C is involved in the transportation of cholesterol to the liver for its catabolism [35]. In ISO-only-treated animals, the HDL-C level remarkably lowered, while serum total cholesterol, TGs, LDL-C, and VLDL-C were significantly elevated. These changes in lipid concentrations might be due to enhanced lipid biosynthesis. Pretreatment with EEOT decreases the levels of serum total cholesterol, TGs, LDL-C, VLDL-C, and elevated HDL-C concentration. Recently, it has been reported that neutraceutical supplements can significantly reduce LDL-C, decrease TGs, and increase HDL-C [36]. The protective effect observed in this study may be due to the potent antioxidant property of the extract of *Citrus medica*, which protects cardiac muscle from the oxidative damage and helps in maintaining the myocardial cell membrane integrity and function thereby protecting the rupture and preventing leakage of the enzyme and lipids [37]. The potent antioxidant activity of hesperidin was shown to have lipid metabolism improving capacity in ISO-induced cardiotoxicity [24]. Oxidative stress is a redox shift in the normal prooxidant/antioxidant balance, and this unbalance may also cause depletion of the antioxidants in the defense system, due to generation of ROS [38]. The administration of ISO produced oxidative stress by inducing lipid peroxidation (LPO) in cardiac muscle.

Elevated LPO is a measure of cell membrane injury along with an alteration in its structure and function [39]. In the current investigation, ISO-treated animals have shown an increased concentration of malondialdehyde (MDA), suggests over-activity of LPO leading to tissue damage and diminishing or preventing antioxidant defensive mechanism causing failure to check excessive free radical formation [40]. However, pretreatment of rats with EEOT significantly diminished cardiac tissue MDA concentration. That might be due to the free radical scavenging activity of the otroj extract. Furthermore, in this study nonprotein sulfhydryl (NP-SH) and total proteins (TP) contents were estimated in the cardiac tissue. It is well established that sulfhydryls are an endogenous antioxidant which are involved in the protection of normal cell structure and function by maintaining redox homeostasis and suppressing of free radicals and by involving in detoxification process [41]. Our study confirmed the earlier findings [42] that elevation in lipid peroxidation is a consequence of depletion of free-SH. The concomitant administration of EEOT significantly restored the NP-SH and total protein concentration in the heart muscle, when compared to the isoproterenol-treated group. The reduction in the total protein content observed in heart tissue of ISO-induced myocardiopathy might be due to inhibition of glycoprotein synthesis [43]. Pretreatment of rats with EEOT prevented the ISO-induced alterations in the levels of protein content in heart tissue. Experiments on *in vitro* antioxidant activity of the EEOT revealed a strong activity in the present investigation and high phenolic content. The possible mechanisms by which phenolic compounds including flavonoids could exert antioxidant activities include direct free radical scavenging capacities due to electron donating ability or indirectly by increasing the capacity of endogenous antioxidant defenses such as increasing production of NO, increasing the concentration of glutathione, activation of antioxidative enzymes, e.g., superoxide dismutase, peroxidase, and catalase or by reducing the formation of endogenous ROS [44,45,46]. In addition to that, cardioprotective activity of phenolic compounds including flavonoids could arise by other mechanisms such as inhibition of signal transducer and activator of transcription 1 (STAT1) [47]. These findings support the observed protective effect of the EEOT on various enzymatic and non-enzymatic parameters. These findings further support the cardioprotective property of EEOT in the histological assessment. Rats, which received EEOT pretreatment, followed by isoproterenol administration, showed minimal histological changes.

## 5. Conclusions

In conclusion, the administration of *Citrus medica* “otroj” extract (EEOT) prevented biochemical and histomorphological alteration induced by ISO. The cardioprotective effect of EEOT could be attributed to the presence of antioxidative phenolic content as well as vitamin C in the fruits of *C. medica*, which cause significantly lowering the oxidative threat and leading to normal physiological function. The findings support the use of *C. medica* fruits as cardiotonic and antioxidant drug. Further work should be embarked upon with a view to elucidate further possible mechanisms of action of the extract. The present results can form the basis for selection of *C. medica* for further investigation in the potential discovery of naturally occurring bioactive compounds. Studies aimed at the isolation and structure elucidation of cardioprotective and antioxidant active constituents from *C. medica* are in progress.
